# How Much Should Consumers with Mild to Moderate Hearing Loss Spend on Hearing Devices?

**DOI:** 10.3390/audiolres15030051

**Published:** 2025-05-05

**Authors:** Vinaya Manchaiah, Steve Taddei, Abram Bailey, De Wet Swanepoel, Hansapani Rodrigo, Andrew Sabin

**Affiliations:** 1 Department of Otolaryngology–Head and Neck Surgery, University of Colorado School of Medicine, Aurora, CO 80045, USA; dewet.swanepoel@up.ac.za; 2 UCHealth Hearing and Balance, University of Colorado Hospital, Aurora, CO 80045, USA; 3 Virtual Hearing Lab, Collaborative Initiative Between University of Colorado School of Medicine and University of Pretoria, Aurora, CO 80045, USA; 4 Department of Speech-Language Pathology and Audiology, University of Pretoria, Pretoria 0002, South Africa; 5 Department of Speech and Hearing, School of Allied Health Sciences, Manipal Academy of Higher Education, Manipal, Karnataka 576104, India; 6 Hear Advisor LLC, Rockford, IL 61102, USA; steve@hearadvisor.com (S.T.); abram@hearingtracker.com (A.B.); andy@hearadvisor.com (A.S.); 7 Hearing Tracker Inc., Austin, TX 40702, USA; 8 School of Mathematical and Statistical Sciences, University of Texas Rio Grande Valley, TX 78539, USA; hansapani.rodrigo@utrgv.edu

**Keywords:** hearing aids, sound quality, hearing aid price, direct-to-consumer hearing aids, over-the-counter hearing aids, consumer metric

## Abstract

**Background:** This study examined the relationship between hearing device price and sound quality. **Method:** A novel consumer-centric metric of sound quality (“SoundScore”) was used to assess hearing devices’ audio performance. Each hearing device is tested with two fittings. The “Initial Fit” is designed to approximate the most likely fitting for an individual with a mild-to-moderate sloping sensorineural hearing loss. The “Tuned Fit” includes adjusting parameters optimized to hit prescriptive fitting targets (NAL NL2) on an acoustic manikin. Each fitting is evaluated across five dimensions. Both fittings are combined using a weighted average to create a single number from 0 to 5 representative of a device’s overall audio performance. Seventy-one hearing devices were tested. **Results:** A strong positive correlation was found between hearing device price and SoundScore. The average SoundScore increased dramatically as the price approached USD 1000, with marginal improvements beyond this point. SoundScore was consistently poor for devices under USD 500, highly variable between USD 500–1000, and consistently good over USD 1000. **Conclusions:** There is a strong but nonlinear relationship between hearing device price and sound quality. This information can aid consumers in making informed decisions while also assisting hearing healthcare professionals in providing comprehensive guidance to their patients.

## 1. Introduction

Hearing devices continue to develop in terms of aesthetics, technological capabilities, and features due to advancements in design and engineering. Recently, regulatory changes in the United States at the Food and Drug Administration (FDA) created a new over-the-counter (OTC) hearing aid category that included two categories of OTC hearing aids—self-fitting over-the-counter hearing aids (OTC-SF) and preset over-the-counter hearing aids (OTC-PS). This expands the available options from the preexisting categories of hearing devices, which include prescription hearing aids (Rx HA) and personal sound amplification products (PSAPs) [1]. The cost of hearing devices can vary substantially between and within hearing device categories. For example, the cost of Rx HAs can range from approximately USD 1000 per pair for entry-level models to USD 8000 for high-end devices. Similarly, OTC-SF hearing aids typically cost between USD 600 and USD 1000, with premium options reaching up to USD 2400. The OTC-PS and PSAPs start around USD 100 and USD 20 a pair, respectively. While this expansion of options has enhanced accessibility, it has also increased demands on the consumer with regard to self-navigating the options and potentially self-selecting an appropriate hearing device.

Reports from popular media suggest that individuals with hearing loss experience confusion about the relationship between price and audio performance [2]. Consumers are faced with the question of how much to invest in their hearing devices. This decision is further complicated by significant price disparities among device categories. For instance, a pair of PSAP devices could cost as low as USD 20, whereas a high-end Rx HA pair could cost as high as USD 8000. While there are often considerable differences in features, functionalities, and performance between these device categories, the devices themselves can appear physically similar and claim to offer similar benefits, yielding growing confusion among consumers.

Several studies have examined the relationship between hearing aid technology level and hearing aid outcomes like benefit and satisfaction [3,4,5,6]. Findings have suggested that higher technology levels, which are typically associated with higher costs, do not yield better hearing aid outcomes. The relationship between technology level and benefit is complicated by the so called “price-quality heuristic”, a cognitive bias which leads people to associate higher-priced products with higher quality. For example, a recent consumer survey showed that price was positively correlated with self-perceived benefit [7]. To our knowledge, no study has directly investigated the link between the price of a hearing device and its overall sound quality performance in everyday scenarios.

In a recent effort, the project from Hear Advisor (https://www.hearadvisor.com/ (accessed on 2 February 2025)) created a novel consumer-centric metric of hearing device audio performance [8,9]. The metric is referred to as “Sound Score”, which is a rating of audio performance in a Likert scale of 1–5, with 5 rating being the highest. It provides a standardized method for assessing how well a hearing aid enhances sound clarity, speech understanding, and overall listening experience in real-world environments. SoundScore was developed by prioritizing evidence-based methods to evaluate different elements of audio performance, making it a valuable tool for both consumers and audiologists when selecting and optimizing hearing devices. By bridging the gap between objective performance metrics and subjective user experience, this approach supports personalized hearing healthcare and helps individuals make informed decisions about their hearing solutions. In a recent study, we reported how SoundScore may vary between different hearing device categories such as the Rx HA, OTC-SF, OTC-PS, and PSAPs [9].

In the current study, we aim to examine the relationship between hearing device price and its overall audio performance using the newly developed consumer-centric metric “SoundScore” [8].

## 2. Method

### Study Design and Data Collection

The details of the procedures underlying SoundScore are presented in on open-source document and a recent publication [8,9]. Each hearing device is tested with two fittings. The “Initial Fit” is designed to approximate the most likely fitting (acoustic coupling and tuning) for an individual with a mild-to-moderate sloping sensorineural hearing loss, N3 configuration [10]. The goal of this step was to achieve a fit that would be based on the manufacturers’ targets and/or methods. For most prescription devices, this means using the manufacturer’s “Initial Fit”, and for non-prescription devices, this usually means completing the onboarding procedure. The device is also tested in a “Tuned Fit” where all adjustable parameters are optimized to be as close as possible to National Acoustic Laboratories–Non-Linear 2(NAL-NL2; [11]) binaural experienced-user targets at 55, 65, and 75 dB SPL input as measured on a Knowles Electronics Manikin for Acoustic Research (KEMAR [12]). Fit was determined by comparing the insertion gain (aided-minus-open ear) to the prescribed targets.

For each fitting, the performance was evaluated across 5 dimensions. The first and second dimensions were the predicted increase to “speech in quiet” (speech intelligibility in quiet-to-moderate noise: <70 dB SPL, avg SNR = 10.4 dB) and “speech in noise” (speech intelligibility in loud noise: >70 dB SPL, avg SNR = 1.2 dB), respectively. These were assessed in 72 ambisonic audio scenes that were created using the 12 environments from the ARTE database [13], each combined with 6 custom talker conditions. The scenes were presented over an 8-channel speaker array to KEMAR wearing the devices. For each scene, speech intelligibility was predicted using the Hearing Aid Speech Perception Index V2 (HASPIv2; [14]). For the metrics of interest here, the increase in HASPIv2 is averaged for quiet-to-moderate and loud (>70 dB SPL) environments. The third metric is “own voice” (i.e., own-voice boominess). This is estimated via the Real Ear Occluded Insertion Gain [15] in most devices. However, when a device has Active Occlusion Cancelation [8], we instead measure objective occlusion via probe mics during vocalization in human participants. The fourth metric is “feedback handling” (i.e., how often the device creates audible feedback). This was not assessed objectively, but rather using blind subjective evaluation of recordings made under conditions of nearby hand movement and hand cupping. Finally, the fifth metric is “music streaming” (i.e., the sound quality of streamed music). This is assessed using the Hearing Aid Audio Quality Index [16] averaged across 5 music genres and played at a calibrated level designed to represent a typical music listening level. For more methodological detail on all metrics, see our prior publications [8,9].

The values of each of the five metrics were combined to a single number per fit (i.e., Initital Fit and Tuned Fit) using the average importance scores computed from a survey of hearing aid professionals and consumers who were required to rank the order of importance of each metric. These scores applied a high weighting to the two speech intelligibility metrics (0.34 and 0.29 for speech in quiet and loud, respectively) and low weighting to the other metrics (feedback: 0.12, own voice: 0.15, music quality: 0.10). Finally, the two fit scores were combined into a single score. This was carried out by using weightings inferred from a survey of 257 hearing-aid consumers asking them to value the importance of a device needing minimal effort to sound good. These scores apply a high weighting to the Initial Fit (0.77) and a low weighting to the Tuned Fit (0.23). The resulting value is a single number from 0 to 5 termed the “SoundScore.” All of these data, including the hearing device brand and model, are available in the HearAdvisor website (www.hearadvisor.com (accessed on 2 February 2025)).

This procedure was run on seventy-one hearing devices (Rx HA = 16, OTC-SF = 11, OTC-PS = 34, PSAPs = 10) priced between USD 69 and USD 7103 per pair for consumers in the United States. All the data available publicly in the HearAdvisor website at the time of this study was included. For non-prescription products, prices were determined using the values on retailer websites. For prescription products, the average price reported by professional users of HearingTracker.com was used. It is important to recognize that the price of prescription devices also includes the cost of services from the clinician. The total price was used here instead of the cost of the device (i.e., price without any services). This is because it is very hard to separate these two aspects as each setting may purchase devices at different price and may also have a different percentage of service charges, making this separation extremely complex. Nevertheless, what it costs to the user is often what influences consumer decision-making. For this reason, we believe this is a reasonable approach.

## 3. Data Analysis

All analyses were performed with R statistical software (Version 4.2.2). The data violated the assumptions of normality and homogeneity of variance. For this reason, the differences in SoundScore across hearing device categories based on price were assessed using the non-parametric Kruskal–Wallis rank sum test. The post hoc pairwise differences were assessed using Wilcoxon rank sum tests with Benjamini–Hochberg (BH) adjustment for multiple comparisons. *P*-values provided for all post hoc comparisons are BH-adjusted. Spearman’s correlation was performed to examine the relationship between different elements of sound quality ratings and SoundScore. All tests were two-tailed and performed at a threshold of 5% level of significance.

## 4. Results

**Figure 1:**
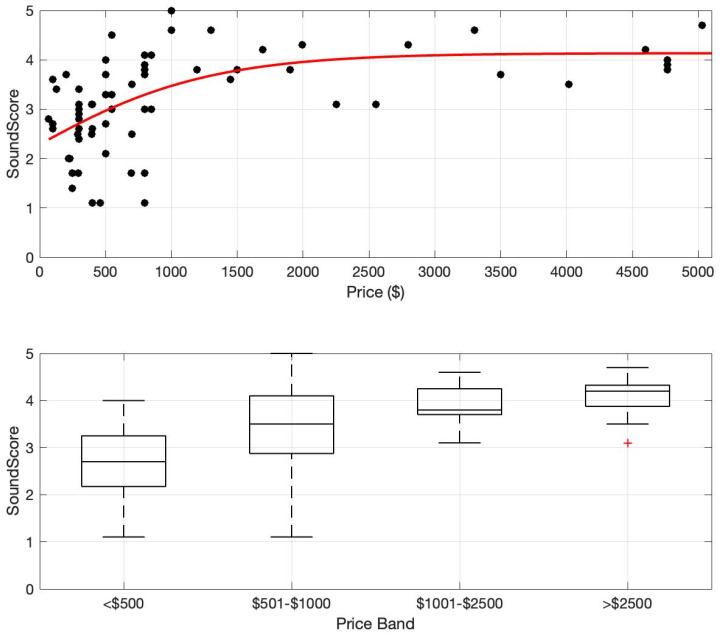
SoundScore vs. price. (**Top**) SoundScore and price values shown for 73 hearing devices (circles) and a trend line (third-order polynomial) fitted to the full group. (**Bottom**) Boxplots of SoundScore distributions for four different price bands. Box shows the middle 50% of the distribution, whiskers show the distribution limits, and the horizontal line shows the median.

The SoundScore of all hearing devices as a function of price is plotted in Figure 1 (Top). There is a clear trend where SoundScore increases with price and then saturates. The saturation occurs in the USD 1000–1500 range. To further aid analysis, the devices were separated into four price bands: <USD 500, USD 501–1000, USD 1001–2500, and >USD 2500. The price band was derived to differentiate different hearing device categories (e.g., PSAP and OTC-PS hearing aids are generally priced below USD 500, OTC-SF hearing aids are priced between USD 500 and 1000, entry- and mid-level Rx HA are priced between USD 1000 and 2500, and premium Rx HA are priced over USD 2500).

The median and interquartile ranges of SoundScore for each price band are shown in Figure 1 (Bottom). A non-parametric Kruskal–Wallis rank sum test was performed to examine the differences in sound scores of hearing devices across four price categories: <USD 500, USD 501–1000, USD 1001–2500, and >USD 2500. The results revealed a significant difference in sound scores among these groups (*χ*^2^ = 32.974, *df* = 3, *p* < 0.001), suggesting that price category influences audio performance. The post hoc analysis revealed that the sound scores differed significantly among several price categories. The <USD 500 category exhibited significantly lower sound scores compared to the USD 501–1000 (*p* = 0.018), USD 1001–2500 (*p* = 0.0007), and >USD 2500 (*p* < 0.001) categories. Additionally, sound scores in the USD 501–1000 range were significantly lower than those in the >USD 2500 category (*p* = 0.041). However, there were no significant differences between the devices in the USD 501–1000 and USD 1001–2500 categories (*p* = 0.27) or between the USD 1001–2500 and >USD 2500 (*p* = 0.27) categories, suggesting that sound quality improves more at lower price points but stabilizes at higher price ranges. 

Correlations between price and each of the component metrics that make up SoundScore were also examined using Spearman’s correlation coefficients (see Table 1). SoundScore has the strongest positive correlation with hearing device price. Speech in quiet and overall score per fit had a strong positive correlation with hearing device price. Speech in noise and own voice had a moderate positive correlation to price. Feedback handling had a moderate negative correlation with price, suggesting the higher the hearing device price, the smaller the feedback issues. Within the feedback domain, the higher the score, the better the performance. Finally, the music streaming metric had a weak positive correlation with price. Overall, these results suggest that performance on most component metrics improves with hearing device price, except for feedback handling.

## 5. Discussion

To our knowledge, this study is the first to directly investigate the relationship between the price of contemporary hearing devices and their overall audio performance. Overall, this study suggests an increasing but saturating relationship between hearing device price and audio performance. The data indicate that the audio performance of the tested hearing devices reaches saturation around USD 1000–USD 1500 per pair (Figure 1, Top). In the current marketplace, this value might be a helpful minimum for consumers to consider when seeking the highest performing hearing assistance devices at a reasonable cost. It is also important to recognize that hearing device selection is multifaceted, with audio performance being one of several important factors—other factors to consider include comfort, usability, durability, connectivity, rechargeability, power consumption, etc. [17].

In a related study using the same method, the relationship between SoundScore among different hearing device categories [9] was examined. This study showed significant differences among SoundScore across hearing device categories, with Rx HA having the highest median score, followed by OTC-SF and OTC-PS, and the PSAPs having the lowest median scores. These device categories are differentiated by features and functionalities, as well as their retail price, with Rx HA having the highest price and PSAPs having the lowest price, on average. Nevertheless, the price of the device can also vary substantially within each device category, as noted in the Introduction section. As discussed earlier, understanding the distinctions between these device categories and identifying their key benefits and limitations can be challenging for consumers. Some consumers, especially when purchasing hearing devices as a gift for their significant other, may opt to purchase cheaper devices such as PSAPs [18].

The current study results emphasize that hearing device price and audio performance is related, which could impact users’ experiences and outcomes (Bannon et al., 2023 [7]). Studies evaluating the acoustic performance of hearing devices using traditional electroacoustic methods and the clinical trials that have examined the efficacy of direct-to-consumer (DTC) devices have shown varied results. For instance, the earliest peer-reviewed publication that evaluated the electroacoustic performance of OTC hearing aids in Hong Kong suggested that most of the devices evaluated were of poor acoustic quality and do not meet the prescription gain requirements for individuals with hearing loss [19]. In contrast, some laboratory studies evaluating the performance of PSAPs [20] and some recent clinical trials examining the outcomes of OTC hearing aids [21] have shown positive outcomes of these devices for individuals with hearing loss. One explanation for these differences in study outcomes is the possible device sampling bias in clinical trials as researchers have ensured acoustically good quality device in these studies [1]. As demonstrated in our recent study on SoundScore differences across hearing device categories, there are high audio-quality DTC devices, including OTC-SF, OTC-PS and some PSAPs [9]. However, there is significant variability in audio performance among the devices currently available on the market. Therefore, further studies are necessary to explore the relationship between hearing device price and factors such as user acceptance, expectations and outcomes.

In general, hearing device price is linked to the level of technology, with higher-priced hearing aids typically featuring more advanced technology, and vice versa. Therefore, price can often serve as a proxy for the level of technology. As highlighted earlier, studies evaluating speech quality in laboratory settings [22] and assessing hearing aid benefit and satisfaction [4,5] have shown that these outcomes do not significantly differ between basic versus premium hearing aids.

In the current study, there was greater variation in the level of hearing device technology among DTC hearing devices. Specifically, OT-PS hearing aids and PSAPs generally have fewer features and functionalities compared to OTC-SF hearing aids and prescription hearing aids. Consequently, there is a need for high-quality clinical trials that directly compare different categories of hearing devices to understand how these variations in technology impact user outcomes. Such studies would be particularly timely and valuable. Moreover, our findings indicate that optimal audio performance is achieved in devices with a price point of approximately USD 1000–USD 1500 per pair for individuals with mild-to-moderate hearing loss in the United States. As most health insurances do not cover hearing aids, this could be an unaffordable out-of-pocket cost for many individuals who need these devices [23]. In light of these findings, efforts are needed to develop more affordable hearing device solutions that offer high sound quality and audio performance [24]. This would further increase accessibility to effective hearing assistance for a wider range of consumers.

## 6. Study Limitations and Future Directions

The study results should be considered in light of several limitations. First, to ensure standardization, a single audiometric pattern of mild-to-moderate hearing loss was used, which oversimplifies the range of hearing loss profiles. Second, the SoundScore metric only focused on key audio performance elements and does not account for other important factors that consumers might consider when making a decision, such as the appearance of the device, comfort, brand. This is important as, in this study, we focused on hearing device cost as a factor in consumer decision making. Third, the relationship between SoundScore and user performance was not investigated. Fourth, for simplicity, we provided single prices for all devices. However, prescription hearing aids in the US often come with bundled packages that include both device and professional services, which is not the case for DTC hearing devices. Nevertheless, the overall price used is reflective of what the consumer would pay. Finally, the consumer expectations for audio performance may be influenced by how much they spend on a device. For instance, expectations for a USD 100 device may differ significantly from those for a USD 1000 or a USD 5000 device. Further studies are needed to explore the relationship between metrics like SoundScore and hearing aid outcomes such as benefit and satisfaction. Additionally, a health economics perspective is necessary to understand consumer expectations and the value they place on hearing devices relative to their price.

## Figures and Tables

**Table 1:** Spearman’s correlation between SoundScore or its component metrics and hearing device price (*p*-value: * < 0.01; ** < 0.001).

Hearing Device Quality Elements	Correlation with Price (*p*-Value)
SoundScore	0.71 **
Speech in quiet (Initial Fit)	0.59 **
Speech in noise (Initial Fit)	0.51 **
Own voice (Initial Fit)	0.43 **
Music streaming (Initial Fit)	0.39 **
Feedback handling (Initial Fit)	−0.22
Speech in quiet (Tuned Fit)	0.74 **
Speech in noise (Tuned Fit)	0.55 **
Own voice (Tuned Fit)	0.41 **
Music streaming (Tuned Fit)	0.37 *
Feedback handling (Tuned Fit)	−0.38 **

## Data Availability

Data used in this study is freely available in the HearAdvisor website. The raw data supporting the conclusions of this article will be made available by the authors on request.
